# Uncovering myocardial infarction genetic signatures using GWAS exploration in Saudi and European cohorts

**DOI:** 10.1038/s41598-023-49105-1

**Published:** 2023-12-10

**Authors:** Amein K. Al-Ali, Abdullah M. Al-Rubaish, Rudaynah A. Alali, Mohammed S. Almansori, Mohammed A. Al-Jumaan, Abdullah M. Alshehri, Mohammed S. Al-Madan, ChittiBabu Vatte, Tess Cherlin, Sylvia Young, Shefali S. Verma, Grant Morahan, Bobby P. C. Koeleman, Brendan J. Keating

**Affiliations:** 1 Department of Clinical Biochemistry, College of Medicine, Imam Abdulrahman bin Faisal University, 3144 Dammam, Saudi Arabia; 2 Department of Internal Medicine, King Fahd Hospital of the University, 34445 Al-Khobar, Saudi Arabia; 3 College of Medicine, Imam Abdulrahman bin Faisal University, 31441 Dammam, Saudi Arabia; 4 Department of Emergency Medicine, King Fahd Hospital of the University, 34445 Al-Khobar, Saudi Arabia; 5 Department of Pediatrics, King Fahd Hospital of the University, 34445 Al-Khobar, Saudi Arabia; 6 Department of Pathology and Laboratory Medicine, University of Pennsylvania, Philadelphia, PA USA; 7 Centre for Diabetes Research, Harry Perkins Institute of Medical Research, University of Western Australia, Nedlands, 6009 Australia; 8 Department of Genetics, University Medical Center Utrecht, Utrecht, 85500/3508 GA The Netherlands; 9 Department of Surgery, Perelman School of Medicine, University of Pennsylvania, Philadelphia, PA USA

**Keywords:** Biochemistry, Genetics, Cardiology

## Abstract

Genome-wide association studies (GWAS) have yielded significant insights into the genetic architecture of myocardial infarction (MI), although studies in non-European populations are still lacking. Saudi Arabian cohorts offer an opportunity to discover novel genetic variants impacting disease risk due to a high rate of consanguinity. Genome-wide genotyping (GWG), imputation and GWAS followed by meta-analysis were performed based on two independent Saudi Arabian studies comprising 3950 MI patients and 2324 non-MI controls. Meta-analyses were then performed with these two Saudi MI studies and the CardioGRAMplusC4D and UK BioBank GWAS as controls. Meta-analyses of the two Saudi MI studies resulted in 17 SNPs with genome-wide significance. Meta-analyses of all 4 studies revealed 66 loci with genome-wide significance levels of p < 5 × 10^–8^. All of these variants, except *rs2764203*, have previously been reported as MI-associated loci or to have high linkage disequilibrium with known loci. One SNP association in *Shisa family member 5* (*SHISA5*) (rs11707229) was evident at a much higher frequency in the Saudi MI populations (> 12% MAF). In conclusion, our results replicated many MI associations, whereas in Saudi-only GWAS (meta-analyses), several new loci were implicated that require future validation and functional analyses.

## Introduction

Coronary artery disease (CAD) leading to myocardial infarction (MI) is a leading cause of mortality, and modifiable risk factors, including sedentary lifestyle, diet, and smoking, play major roles in disease risk^1^. While exogenous risk factors, including dyslipidaemia, type 2 diabetes (T2D), and hypertension, exacerbate disease progression, 40–60% of CAD susceptibility has been attributed to genetic factors^2–5^. Genome-wide association studies (GWAS) have yielded significant insights into the complex aetiology of CAD and MI, including the interplay of hundreds of genetic risk variants impacting phenotypic development, as well as CAD-independent variants that impact the risk of MI alone^6^. These genetic variants provide important insights into the molecular mechanisms underlying MI and can lead to potential downstream targets for therapeutic intervention. However, much work remains to be done to fully understand the complex interplay between genetic and environmental factors in the development and progression of CAD and MI.

Large international consortia, including the UK Biobank (UKBB), Million Veteran Program (MVP), Coronary ARtery DIsease Genome-Wide Replication and Meta-Analysis (CARDIoGRAM), and Coronary Artery Disease (C4D) Genetics Consortia studies, have provided large-scale population-based cohorts to study the genetic underpinnings of CAD and/or MI^7–12^. However, most study participants in these large consortia are of European ancestry. The need for improved diversity of populations in genomic studies has been recognized, and while some CAD-related GWAS meta-analyses in other ancestral groups have been performed^13–15^, further large-scale studies are needed to evaluate the frequencies and consistency of risk allele effect sizes across different ancestries and to assess linkage disequilibrium, which can vary substantially across genetic ancestries^15^.

Performing GWAS in Saudi Arabian populations offers a unique opportunity to discover novel genetic variants impacting disease risk, as there is a high rate of consanguinity among tribal pedigrees, leading to a higher frequency of rare genetic variants due to increased levels of shared ancestry. Furthermore, undetected or untreated CAD is a significant health and financial burden in Saudi Arabia, with community-based epidemiological studies reporting a prevalence of CAD of approximately 55 cases per 1000 individuals in 30- to 70-year-old adults^16–18^.

In this study, genome-wide genotyping (GWG), imputation and GWAS followed by meta-analysis were performed based on two independent Saudi Arabian studies comprising 3950 MI patients and 2324 non-MI controls. Meta-analyses were then performed with the two Saudi MI studies together with the CardioGRAMplusC4D and UK BioBank GWAS, which comprised an additional 56,278 MI patients and 577,716 non-MI controls.

## Materials and methods

### Patient sampling and phenotyping

#### Saudi MI Study 1

From 2019 to 2020, samples and data from consecutive subjects with MI visiting the Cardiology Clinics, King Fahd Hospital of the University, Al-Khobar, and King Fahd Hospital, Alhafof, Saudi Arabia, were collected for inclusion in this study. Participants ranged in age from 25 to 66 and were clinically diagnosed with MI at the time of recruitment. Clinical diagnosis of MI was derived according to the fourth universal definition of MI^19^. The phenotypic data of all subjects were reviewed by a cardiologist consultant to verify uniformity among sites and eligibility according to study criteria. Eligibility for each of the individual cases was reviewed by the consultant committee and assessed for inclusion. For secondary analyses, T2D and hypertension were defined using WHO criteria; LDL, HDL, total cholesterol and troponin I were determined using Direct LDL-, Ultra HDL-, Cholesterol- and STAT High Sensitive Troponin I-Alinity c Reagent kits (Abbott, Wiesbaden, Germany)^20,21^.

#### Saudi MI Study 2

Details of the MI patients and controls in this Saudi study are described in a 2016 GWAS of CAD/MI by Wakil et al.^22^. Patients with suspected CAD/MI based on coronary angiography and echocardiography (ECG) abnormalities at the Catheterization Centre of King Faisal Heart Institute, King Faisal Specialist Hospital and Research Centre, Riyadh (KFSHRC), Saudi Arabia, were evaluated and represented all five regions of the country. Changes in the biomarkers myoglobin, cardiac troponin T, pro-brain natriuretic peptide and pro-calcitonin were also assessed. Two experienced interventional cardiologists independently reviewed patient records for the presence of ischaemia as per recommendations of the Joint ESC/ACCF/AHA/WHF Task Force for the Redefinition of MI^23^. The exclusion criteria included major cardiac rhythm disturbances, history of cerebral vascular disease, neurological disorder, psychiatric illness, and substance abuse. Controls consisted of individuals from KFSHRC undergoing heart valvular disease surgery and subjects with chest pain but no significant coronary stenosis based on angiography. There were 3481 MI patients available after delineating MI from CAD-alone cases, with 2299 controls.

**Figure 1:**
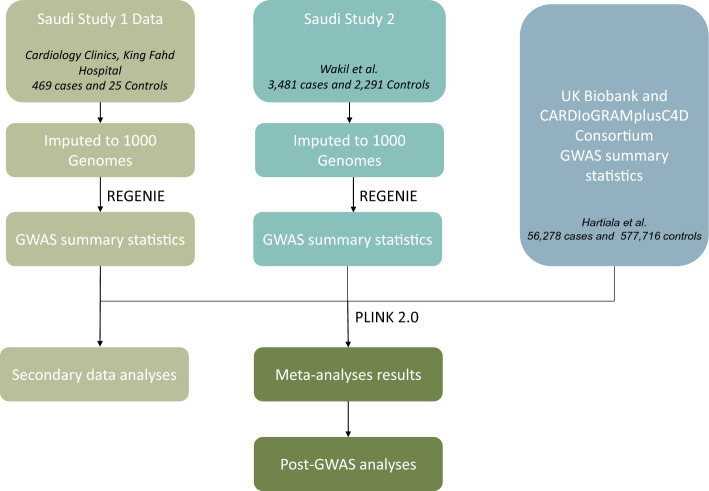
This flowchart provides a visual representation of the study design, detailing the progression from participant recruitment to statistical analyses.

Details regarding the UK Biobank and CARDIoGRAMplusC4D Consortium GWAS MI patients (56,278 subjects), controls (577,716 non-MI subjects), phenotype ascertainment, and ancestry information are described elsewhere^9^. The study design for these analyses and details of how the datasets were combined is also depicted in the flowchart shown in Fig. 1.

For the Saudi MI Study 1, ethical approval was obtained from the Imam Abdulrahman Bin Faisal University Institutional Review Board (IRB) committee (IRB-2019-01-104), and the study was conducted according to the ethical principles of the Declaration of Helsinki and Good Clinical Practice guidelines. Informed written consent in English, with a verified translation in Arabic, was obtained from all participants in accordance with the IRB rules. The Saudi MI Study 2 protocol was approved by the Institutional Review Board (IRB) of the King Faisal Specialist Hospital and Research Centre. Summary-level GWAS datasets for the UK BioBank and CardioGRAMplusC4D were downloaded through a resource database outlined in Hartiala et al.^9^.

### Generation of genotype data and imputation

#### Saudi MI Study 1

Peripheral blood samples were collected in EDTA tubes and stored at 4 °C before extraction of genomic DNA using Gentra Puregene Blood kits (Qiagen, Maryland, USA) according to the manufacturer’s protocol. DNA concentrations and purity were estimated by fluorometry using a NanoDrop 2000 Spectrophotometer (Thermo Fisher, MA, USA) and were diluted to 20 ng/µl. GWG was then performed using the Infinium Global Screening Array v3.0 (Illumina, CA, USA), which captures 654,027 SNPs or monomorphic/rare variants. Genotype data were clustered using Illumina GenomeStudio software, and standard quality control (QC) was performed using PLINK^24^. Normalized intensities for all samples were generated using optiCall clustering^25^. Raw genotypes were imputed using the 1000 Genomes Project (1KGP) v3 multiethnic reference panel through the Michigan Imputation Server^26^. The genotype data were subjected to QC with variants with < 90% missingness and consistency against the Haplotype Reference Consortium (HRC) reference panel for strand, reference/alternative alleles, SNP names and genome build positions. Furthermore, the imputed data were subjected to QC to retain variants with imputation INFO scores of R^2^ > 0.3 using Minimac, a 99% genotyping and sample call rate, and minor allele frequency (MAF) > 0.01^27^. Variants with a Hardy–Weinberg equilibrium (HWE) p value < 1 × 10^−8^ were excluded from the analyses. Principal component analyses (PCA) were computed using the fastPCA module in the eigensoft package^28^. The data points were then projected on the 1KGP populations^29^.

#### Saudi MI Study 2

DNA, GWG and QC are described in detail in Wakil et al.^22^. In brief, GWG was performed using Affymetrix Axiom Genome-Wide “ASI Array” (Asian population) with ~ 537,800 directly genotyped SNPs passing QC filtering. CARDIoGRAMplusC4D and UKBioBank GWAS data and imputation are fully described in Hartiala et al.^9^. This data was also imputed to 1000 Genomes dataset using Michigan Imputation server^26^.

### Statistical analyses

Meta-analyses of GWAS: The variants passing QC for imputed dosage data were used to perform genome-wide association analyses for MI patients and controls. To account for the relatedness in the dataset, the analyses for Saudi studies 1 and 2 were performed using REGENIE^30^. Supplementary Fig. 1 illustrates the Manhattan and QQ plot for Saudi study 1 GWAS analyses. The associations were adjusted for age, sex, and the first 4 principal components. Two GWAS meta-analyses were performed to discover MI loci. First, a meta-analysis of Saudi MI studies 1 and 2 was conducted using PLINK 2.0 as shown in supplementary Fig. 2. Second, a meta-analysis of Saudi MI studies 1 and 2 was performed with the CARDIoGRAMplusC4D and UK Biobank MI datasets using PLINK 2.0^31^.

## Results

### Study population characteristics

Table 1 summarizes the demographic characteristics of the two Saudi cohorts included in this study. In both cohorts, there were more subjects with MI represented compared to controls having no MI. Saudi MI Study 1 included 469 patients (95%) and 25 controls (5%), whereas Saudi MI Study 2 included 3481 (60%) patients and 2299 controls (40%). Overall, there were more men than women represented in the study; the male to female ratio in both cohorts was ~ 70% to 30%. Both sexes were equally represented in the control group of Study 2. Study 1 had a balanced median age of 55 (47, 63) years for the patients and 54 (44, 64) years for the controls, while Study 2 was represented by a larger distribution of ages with a median age of 60 (51, 69) for patients and 48 (35, 59) for controls. BMI measurements were not available in 4–10% of study subjects, but of those measured, the median BMI was slightly higher in Study 1 {29.3 (25.8, 32.7) for the patients and 30.1 (27.4, 35.3) for the controls} than in Study 2 {28.9 (25.6, 32.5) for the patients and 28.6 (24,5, 33.4) for the controls}. In Study 2, the patients with MI had much higher counts of hypertension (81%) than those in Study 1 (33%).

**Table 1:** Demographics of the two Saudi cohorts included in the MI meta-analysis.

	Saudi Group 1		Saudi Group 2	
	MI patients, n = 469	Controls, n = 25	MI patients, n = 3,481	Controls, n = 2,299
Female	108 (23%)	8 (32%)	935 (27%)	1160 (50%)
Male	361 (77%)	17 (68%)	2546 (73%)	1139 (50%)
Age	55 (47, 63)	54 (44, 64)	60 (51, 68)	48 (35, 59)
BMI	29.3 (25.8, 32.8)	20.1 (27.4, 35.3)	28.9 (25.4, 35.3)	28.6 (24.5, 33.4)
BMI unknown	49	2	213	100
Hypertension	154 (33%)	12 (50%)	2803 (81%)	1311 (57%)

### Replication of previously reported MI risk loci

#### GWAS meta-analyses of Saudi MI Studies 1 and 2 only

Meta-analyses of 3950 MI patients and 2324 controls from Saudi MI Study 1 and 2 resulted in 17 SNPs (6 loci) reaching genome-wide significance. The Manhattan plot for Saudi data meta-analyses is shown in Supplementary Fig. 2. Supplementary Table 1 shows the Quality control and Quality assurance metrics for the SNP filtering for: the two Saudi MI studies. The meta-analysis summary statistics of Study 1 and 2 signals for *p* < 0.001 are shown in Supplementary Table 2. We tested for replication of eight MI-associated SNPs from the Wakil et al. original GWAS paper from which Study 2 cases and controls were derived, of which 3 SNPs were of genome-wide significance and 5 additional SNPs had a suggestive p value of < 1 × 10^–5^^22^. Seven out of eight SNPs from Wakil et al. were replicated in this study at the Bonferroni threshold (p value ≤ 0.05/8 = 0.006)^22^. The loci for these SNPs are linked to the genes *RNF13 (rs41411047), PDZD2 (rs32793), ITGA1 (rs16880442), CDKN2A/B (rs2891168, rs10757274 and rs1333045), EIF4A3 (rs7211079), KCNE2 (rs998261), NDST2 (rs4691), and MRPS6 (rs28451064)*.

**Figure 2:**
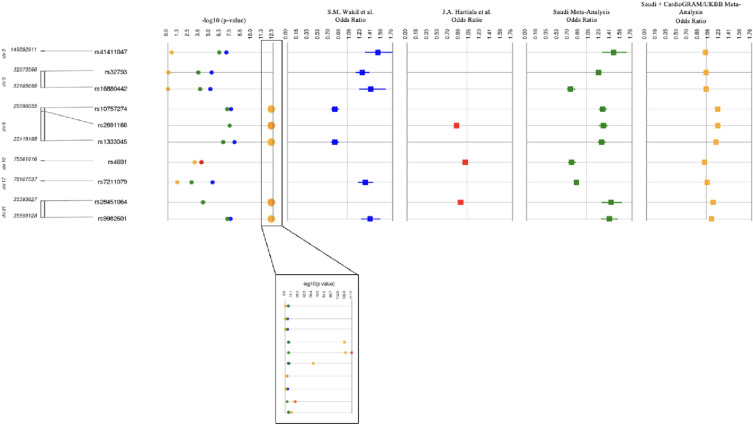
Meta-analysis overview of Saudi MI Study 1 and 2 plus CARDIoGRAMplusC4D + UKBiobank GWAS: Synthesis view plot showing p values from the four analyses in the first panel and their odds ratio and confidence intervals for: Saudi MI Study 2 (Panel 2, blue); CARDIoGRAMplusC4D + UKBioBank (Panel 3, red); Saudi MI Study 1 + 2 (Panel 4, green) and Saudi MI Study 1 + 2 and CARDIoGRAMplusC4D + UKBioBank (Panel 5, yellow). The 10 replicated SNPs are shown on the y-axis.

We also assessed the replication of 213 SNPs with genome-wide significance from the CARDIoGRAMplusC4D + UKBiobank meta-analysis by Hartiala et al.^9^. Three out of 213 SNPs from the Hartiala et al. study demonstrated replication in Saudi data 1 and data 2 meta-analyses^9^. Figure 2 also shows the three SNPs that were replicated from the 213 genome-wide significant SNPs from the Hartiala et al.^9^ meta-analysis. SNPs were considered significant for inclusion if they passed the Bonferroni calculation (p ≤ 0.05/213 = 0.0002).

#### GWAS meta-analyses of Saudi datasets + CardiogramplusC4D + UkBioBank

**Figure 3:**
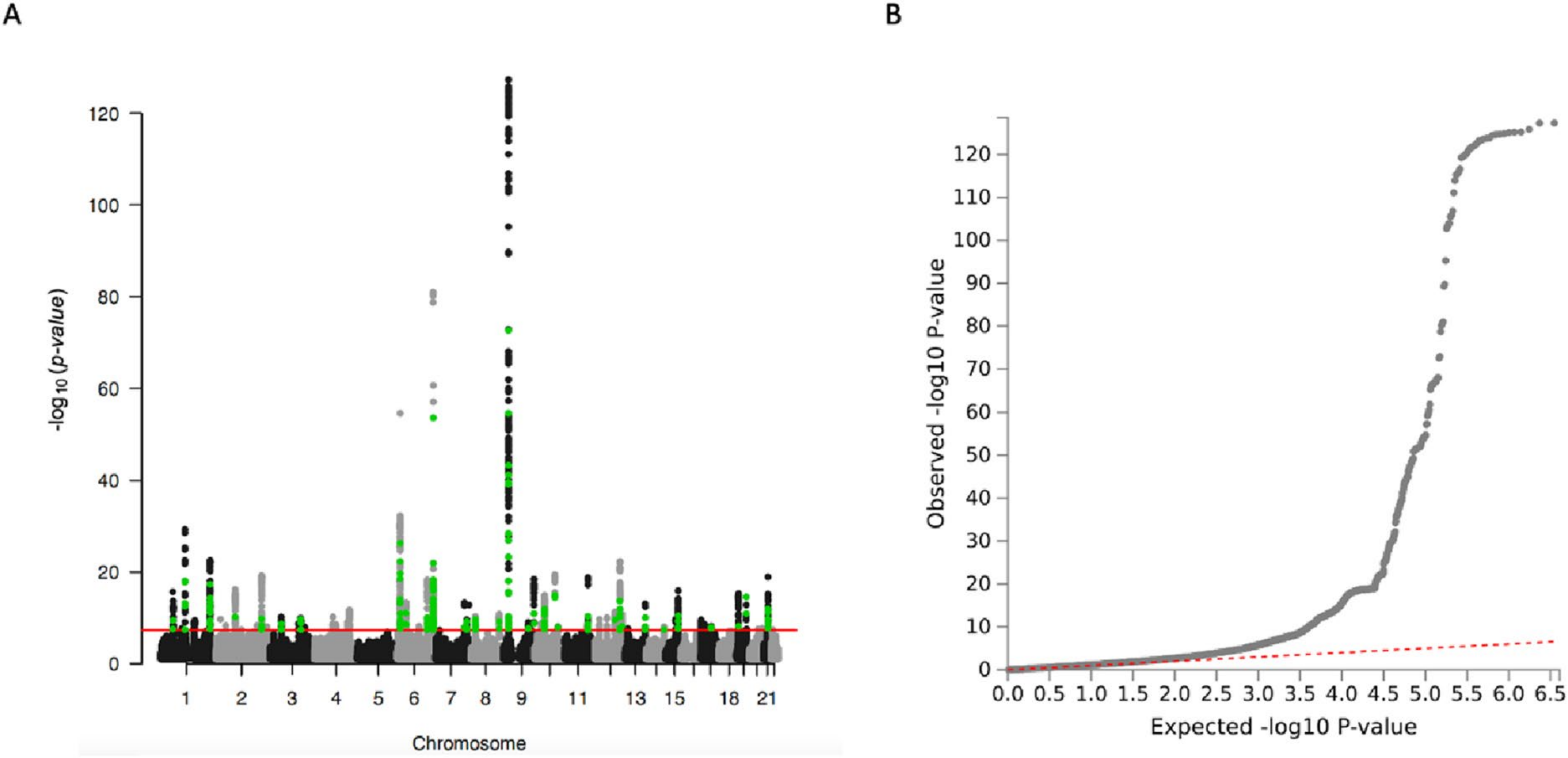
(**A**) Manhattan plot for MI genome-wide significant signals for the full meta-analysis comprising 3950 Saudi MI patients and 2324 controls and 56,278 MI patients and 577,716 controls from CARDIoGRAMplusC4D + UKBiobank. (**B**) Quantile‒Quantile (Q‒Q) plot for the meta-analyses (genomic inflation factor λ = 1.203). The horizontal red line indicates genome-wide significance (p value ≤ 5 × 10–8). SNPs coloured green have not been identified in previous studies.

Figure 3 shows a Manhattan plot for 2523 association signals corresponding to 66 loci (mapping to 212 genes) observed above genome-wide significance (p < 5 × 10–8). The summary statistics of the Saudi MI Study 1 and 2 plus CARDIoGRA MplusC4D + UKBiobank GWAS for p < 0.001 are shown in Supplementary Table  3. The difference in the allele frequencies for all variants in these 66 loci among European and Saudi populations is reported in Supplementary Table 4. Fifteen variants showed a > 10% difference in allele frequencies, but the majority of the variants were common (> 10% MAF) in both populations. Notably, rs11707229 in SHISA5 has an MAF of 0.02 in European populations but an MAF of 0.12 in our Saudi MI populations. The results for all 66 significant genome-wide loci are reported in Table 2. Sixty-five out of 66 loci have been previously implicated to be significantly associated with MI based on the GWAS catalogue (downloaded on April 27, 2023). rs2764203 was previously identified to be nominally associated with MI (p = 1.0 × 10^−7^) but was found to be significantly associated with MI after the addition of the Saudi data in the meta-analyses (p = 2 × 10^–8^).

**Table 2:** The resulting 66 genomic risk loci from GWAS meta-analyses across 60,228 MI patients and 580,040 non-MI controls from Saudi MI Study 1 & 2, the CardioGRAMplusC4D and the UK BioBank.

rsID	Locus	Nearest gene	P value	nGWAS SNPs	nLead SNPs	LeadSNPs	Previously reported SNP in locus	P value (previously reported nearest SNP)
*rs34232196*	chr1:55487346–55496039	*BSND/PCSK9*	3.06E−08	5	1	rs34232196	rs11206510	1.00E−08
*rs9970807*	chr1:56912610–57020650	*PPAP2B*	2.06E−14	55	2	rs9970807; rs41487448	rs17114046	3.00E−15
*rs12743267*	chr1:95212835–95288851	*LINC01057*	2.36E−08	18	1	rs12743267	*rs12743267*	1.00E−08
*rs12740374*	chr1:109776285–109822166	*CELSR2*	5.70E−30	25	2	rs17035665; rs12740374	*rs12740374*	8.00E−32
*rs11810571*	chr1:151706366–151780728	*TDRKH*	6.81E−10	36	1	rs11810571		
*rs6686750*	chr1:154395125–154428505	*IL6R*	3.65E−10	27	1	rs6686750	*rs11810571*	1.00E−09
*rs35700460*	chr1:222720697–222950389	*MIA3*	3.05E−23	209	1	rs35700460	rs2378584	6.00E−35
*rs16986953*	chr2:19942473–19942473	*#N/A*	2.51E−10	1	1	rs16986953	*rs16986953*	2.00E−13
*rs13402621*	chr2:43450843–43578	*THADA:AC010883.5*	5.55E−09	35	1	rs13402621	*rs13402621*	1.00E−09
*rs10176176*	chr2:85,714745–85814984	*RN7SL830P*	7.04E−17	95	1	rs10176176	*rs2886722*	6.00E−20
*rs6761276*	chr2:113830563–113838652	*IL1F10*	3.72E−08	23	1	rs6761276	*rs6761276*	3.00E−08
*rs12693302*	chr2:183182776–183263753	*PDE1A*	5.40E−09	35	1	rs12693302	*rs12693302*	3.00E−09
*rs72926767*	chr2:203243342–204435946	*WDR12:CARF*	6.58E−20	301	1	rs72926767	*rs72926767*	1.00E−21
*rs2161967*	chr2:218667372–218683154	*TNS1*	4.57E−08	14	1	rs2161967	*rs2161967*	2.00E−08
*rs2972149*	chr2:227026531–227172396	*#N/A*	1.00E−08	15	1	rs2972149	*rs2972149*	2.00E−08
*rs73078367*	chr3:48517349–49900350	*NCKIPSD/SHISA5*	7.51E−11	41	3	rs11707229; rs73078367; rs73079003	*rs73078367*	5.00E−11
*rs3772800*	chr3:124438586–124482494	*KALRN*	2.07E−09	9	1	rs3772800	*rs3772800*	8.00E−10
*rs9865841*	chr3:135798658–136669079	*#N/A*	1.28E−10	310	1	rs9865841	*rs9865841*	2.00E−10
*rs2279241*	chr3:138052754–138129143	*MRAS*	4.36E−10	26	1	rs2279241	rs185244	2.00E−13
*rs789294*	chr3:153778760–154088411	*GPR149*	1.18E−08	173	1	rs789294	rs433903	2.00E−08
*rs10857147*	chr4:81158545–81202048	*FGF5*	2.33E−09	12	1	rs10857147	rs16998073	2.00E−12
*rs11099493*	chr4:82576859–82625720	*RASGEF1B:RP11-689K5.3*	7.76E−11	1	1	rs11099493	*rs11099493*	5.00E−11
*rs2452009*	chr4:95447259–95595308	*PDLIM5*	2.92E−09	50	1	rs2452009	rs2452009	6.00E−09
*rs10305839*	chr4:148229662–148427503	*EDNRA*	9.00E−10	61	2	rs4593108; rs10305839	rs72957606	3.00E−21
*rs72689147*	chr4:156614184–156683485	*GUCY1A3*	2.04E−12	40	1	rs72689147	rs11731886	6.00E−13
*rs9349379*	chr6:12718156–13124594	*PHACTR1*	2.21E−55	317	3	rs1412747; rs9349379; rs62389460	*rs9349379*	4.00E−63
*rs2764203*	chr6:34548206–34831761	*RP3-375P9.2*	2.80E−08	93	1	rs2764203	rs2764203	1.00E−07
*rs56336142*	chr6:39124448–39189361	*KCNK5*	5.21E−14	44	2	rs56336142; rs733701	rs1155347	2.00E−16
*rs9486719*	chr6:96841762–97067047	*FHL5*	7.14E−09	180	1	rs9486719	rs9486719	7.00E−10
*rs2327426*	chr6:134098184–134227223	*RP3-323P13.2*	6.05E−19	130	2	rs2327426; rs2327433	rs12190287	3.00E−25
*rs10455872*	chr6:160248806–161682569	*LPA*	9.69E−82	548	14	rs8191728; rs688359; rs3822842; rs2297374; rs9456508	*rs10455872*	4.00E−58
*rs11556924*	chr7:129632081–129685597	*RP11-306G20.1: ZC3HC1*	4.95E−14	3	1	rs11556924	rs11556924	6.00E−14
*rs35586793*	chr7:139714607–139761248	*PARP12*	2.74E−10	43	1	rs35586793	rs35586793	2.00E−10
*rs3918226*	chr7:150690176–150690176	*NOS3*	1.93E−13	1	1	rs3918226	rs3918226	4.00E−13
*rs7011846*	chr8:19759670–19943308	*LPL*	5.63E−11	336	3	rs7011846; rs76722925; rs13276972	*rs7011846*	1.00E−10
*rs2954021*	chr8:126475770–126507389	*RP11-136O12.2*	1.52E−11	52	1	rs2954021	rs2954021	3.00E−13
*rs4977574*	chr9:21693409–22125913	*#N/A*	4.67E−128	464	9	rs11523031; rs117197971; rs13288666; rs3731239; rs36228834; rs28557075	rs2891168	2.00E−141
*rs1967604*	chr9:110505424–110546149	*#N/A*	1.10E−09	45	1	rs1967604	rs1970014	2.00E−11
*rs2519093*	chr9:136132908–136184798	*#N/A*	3.76E−19	42	1	rs2519093	rs2519093	5.00E−19
*rs2505083*	chr10:30300787–30335520	*KIAA1462*	1.94E−09	16	1	rs2505083	rs1887318	4.00E−12
*rs589655*	chr10:44435246–44800379	*RP11-20J15.2*	1.44E−15	393	2	rs1870635; rs589655	rs589655	2.00E−17
*rs1412445*	chr10:91002804–91014061	*LIPA*	3.55E−20	18	1	rs1412445	rs1412445	1.00E−20
*rs17115100*	chr10:104504564–105059896	*CYP17A1*	6.70E−09	126	2	rs17115100; rs79780963	rs11191447	5.00E−16
*rs61908736*	chr11:100520680–100612604	*CTD-2383M3.1*	4.72E−08	30	1	rs61908736	rs61908736	8.00E−09
*rs2019090*	chr11:103524968–103763638	*RP11-563P16.1*	1.66E−19	136	1	rs2019090	rs2019090	2.00E−18
*rs10841443*	chr12:20158160–20247540	*RP11-664H17.1*	2.02E−10	30	1	rs10841443	rs10841443	9.00E−12
*rs7137258*	chr12:54512164–54531481	*RP11-834C11.3: RP11-834C11.5*	9.00E−11	7	1	rs7137258	rs75160195	7.00E−13
*rs2681472*	chr12:89825925–90091782	*#N/A*	5.42E−12	95	1	rs2681472	rs2681472	1.00E−12
*rs10774625*	chr12:111708458–112985328	*#N/A*	4.72E−23	481	1	rs10774625	rs10774625	3.00E−23
*rs1169288*	chr12:121380544–121455873	*HNF1A-AS1:HNF1A*	1.52E−11	51	1	rs1169288	rs1169288	2.00E−12
*rs11057837*	chr12:125303254–125316743	*SCARB1*	2.20E−08	9	1	rs11057837	rs11057837	4.00E−09
*rs9591012*	chr13:32996332–33381342	*N4BP2L2*	2.54E−08	269	1	rs9591012	rs9591012	4.00E−08
*rs11617955*	chr13:110788441–111049623	*#N/A*	1.03E−13	62	5	rs11617955; rs9521632; rs11619113; rs4773141;rs9515203	rs11617955	4.00E−14
*rs7145262*	chr14:100110120–100184101	*HHIPL1*	8.23E−09	42	1	rs7145262	rs9788497	3.00E−14
*rs72743461*	chr15:67441750–67468285	*SMAD3*	2.43E−10	21	1	rs72743461	rs72743461	5.00E−12
*rs7173743*	chr15:78942349–79169499	*MORF4L1*	1.15E−16	361	2	rs62012629; rs7173743	rs7173743	2.00E−17
*rs2760740*	chr17:2015612–2213409	*SMG6*	3.27E−10	247	1	rs2760740	rs4790881	3.00E−11
*rs11652894*	chr17:17698254–18029857	*GID4*	2.96E−09	297	1	rs11652894	rs11652894	5.00E−09
*rs62076439*	chr17:47079416–47513711	*ZNF652*	8.11E−09	122	3	rs4643373; rs62076439; rs55714120	rs62076439	1.00E−09
*rs112374545*	chr19:11159076–11210912	*#N/A*	6.15E−16	88	1	rs112374545	rs6511720	8.00E−22
*rs41290120*	chr19:45319631–45396665	*PVRL2*	2.72E−15	7	2	rs41290120; rs157582	rs7412	5.00E−19
*rs34633566*	chr19:46219145–46374916	*RSPH6A*	2.82E−08	37	1	rs34633566	rs34633566	3.00E−08
*rs6102343*	chr20:39662225–39953467	*ZHX3*	3.44E−08	5	1	rs6102343	rs6102343	2.00E−08
*rs259979*	chr20:57683530–57784527	*ZNF831*	2.34E−08	111	1	rs259979	rs259979	1.00E−08
*rs28451064*	chr21:35586723–35717962	*#N/A*	1.36E−19	118	1	rs28451064	rs28451064	3.00E−22
*rs57636940*	chr22:24636393–24888192	*SPECC1L:SPECC1L-ADORA2A*	3.59E−08	160	1	rs57636940	rs180803	7.00E−10

*nGWAS SNPs refer to the number of GWAS significant SNPs in the loci, and nLead SNPs refer to the number of independent Lead SNPs in the loci. The chromosome start–end positions for risk loci are shown in the locus column. Previously reported SNPs were identified using the *LD trait tool*, and the results from the EBI GWAS catalogue downloaded on 04/05/2023 were used.

## Discussion

We performed GWG, imputation and GWAS on two independent Saudi Arabian studies comprising a total of 3950 MI patients and 2324 non-MI controls. Meta-analyses were performed with the two Saudi MI studies separately, resulting in 6 loci with genome-wide significance, and then combined with the CardioGRAMplusC4D and UK BioBank GWAS SNRPC studies, resulting in 66 loci with genome-wide significance. Our results replicated many MI associations, whereas in Saudi-only GWAS (meta-analyses), several new loci were implicated that require future validation and functional analyses.

The new genome-wide signal for MI from the meta-analyses of the four MI studies, *rs2764203*, is located approximately 4 kb from *RP3-375P9.2* and ~ 20 kb from small nuclear ribonucleoprotein polypeptide C (SNRPC). Very little information is available from any previous studies of the long noncoding RNA *RP3-375P9.2,* apart from an association in a hepatocellular carcinoma (HCC) genomic and epigenomics study within early- and late-stage patients^32^. The *RP3-375P9.2* lncRNA does not appear to be associated with MI in a recent pathway-based study^33^.

Small nuclear ribonucleoprotein polypeptide C (SNRPC) encodes one of the specific protein components of the U1 small nuclear ribonucleoprotein (snRNP) particle, which is needed for the formation of the spliceosome^34,35^. It is critical to the initiation and regulation of pre-mRNA splicing and is broadly expressed in most tissues, including heart tissues^36^. A recent study by Zhang et al. showed that SNRPC has the potential to promote the motility of hepatocellular carcinoma (HCC) cells via induction of epithelial-mesenchymal transition and to serve as a prognostic biomarker in HCC and predictor of immunotherapy responses^37,38^. SNRPC has also been shown to impact sex biases in systemic autoimmune diseases^39^.

The *Shisa family member 5* (*SHISA5*) intronic association (rs11707229) in this MI study is interesting, as the observed minor allele frequency was > 12% in our overall Saudi population but has been reported to be approximately 2% in European populations, less than 1% in African populations and very rare in most Asian populations (http://www.ncbi.nlm.nih.gov/snp/rs11707229). *SHISA5* is a member of the Shisa family, which is a single-transmembrane protein characterized by N-terminal cysteine-rich domains and proline-rich C-terminal regions. *SHISA5* is located in the endoplasmic reticulum and the nuclear membrane and appears to have roles in numerous biological processes including regulation of autophagy, with involvement in p53-inducible pro-apoptosis in a caspase-dependent manner, is inducible by interferon and has an effect on the Wnt signalling pathway^40–43^. Associations of SHISA5 to date are largely limited to anthropometric, red cell characteristics and the glomerular filtration rate (GFR)^44–46^. Lakota and colleagues have previously described the upregulation of SHISA5 in mesenchymal stem cells (MSCs) transplanted into human subjects with ischaemic cardiomyopathy and controls and postulated that SHISA5 contributes to the death of cardiomyocytes via apoptosis after ischaemia–reperfusion injury^47,48^. Alternative splicing isoforms of different C-terminal isoforms of *Shisa5* have been previously reported, and numerous variants impacting alternative splicing acceptor or donor sites appear likely to affect the specificity of its interactions^41^.

In conclusion, our study not only successfully replicated many known MI associations but also, through our Saudi-specific GWAS meta-analyses, identified several novel loci. These newly implicated loci, including *RP3-375P9.2 lncRNA and the SNRPC* gene, present exciting opportunities for future validation and functional analyses. Moreover, the association with SNPs in *SHISA5*, considering the distinct minor allele frequency differences between Saudi and European populations, offers potential insights into the high MI prevalence in Saudi Arabia. Such findings emphasize the critical need for genetic studies across diverse ancestral cohorts to ensure a holistic understanding of MI. This study has numerous limitations, including a limited number of MI controls, discordance in hypertension prevalence between the two Saudi MI studies and incomplete BMI measurements for a small number of the study subjects. Consanguineous populations such as the Saudi Arabian population offer an invaluable opportunity to explore rare and structural variants that are linked to disease. Future studies will involve more elegant methodologies to enhance the power of GWAS in consanguineous populations, inclusion of modifiable and nonmodifiable risk factors in predicting the risk of common diseases and strategic tools to analyse multiple genetic variants and exposure variables to uncover the hidden heritability of MI and concomitant comorbidities.

### Supplementary Information

Supplementary Figure 1.

Supplementary Figure 2.

Supplementary Table 1.

Supplementary Table 2.

Supplementary Table 3.

Supplementary Table 4.

## Data Availability

The datasets generated during the current study are available in the European Variation Archive (EVA) repository (https://www.ebi.ac.uk/ebisearch/search?query=PRJEB59353&submit=Search&db=allebi&requestFrom=global-masthead) under the title "*Genome-Wide Association Studies of Myocardial Infarction in Saudi Arabian Cohorts*" with accession number PRJEB59353.
