# Supplementary figures and images for: Uncovering myocardial infarction genetic signatures using GWAS exploration in Saudi and European cohorts
Source: Sci Rep. 2023 Dec 10;13:21866. doi: 10.1038/s41598-023-49105-1 (PMC10711020; doi:10.1038/s41598-023-49105-1)

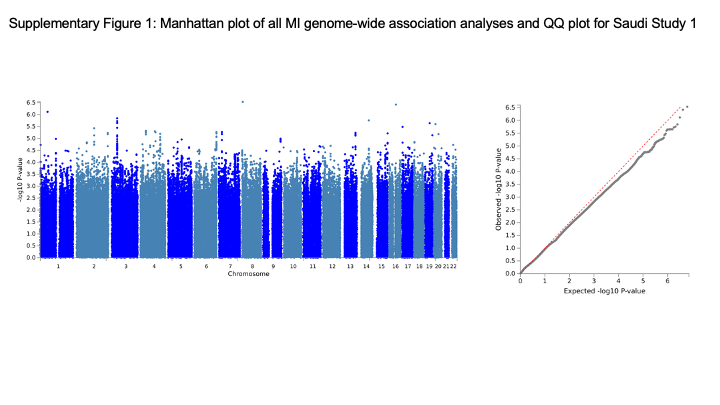

Supplement: Supplementary file 1 — Supplementary Figure 1. [file 41598_2023_49105_MOESM1_ESM.png]

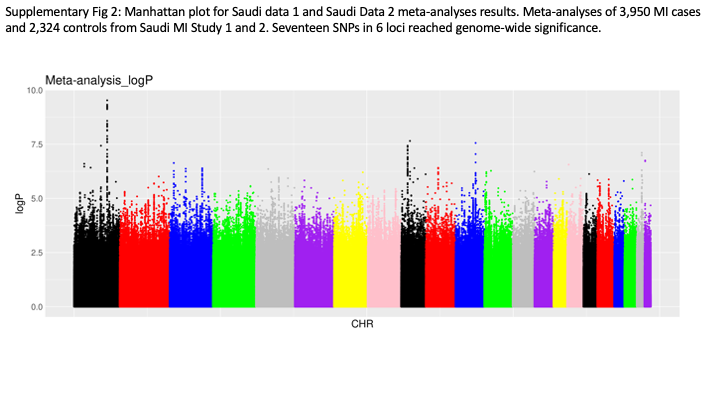

Supplement: Supplementary file 2 — Supplementary Figure 2. [file 41598_2023_49105_MOESM2_ESM.png]
