# Supplementary material for: Uncovering myocardial infarction genetic signatures using GWAS exploration in Saudi and European cohorts
Source: Sci Rep. 2023 Dec 10;13:21866. doi: 10.1038/s41598-023-49105-1 (PMC10711020; doi:10.1038/s41598-023-49105-1)
Supplement: Supplementary file 3 — Supplementary Table 1. [file 41598_2023_49105_MOESM3_ESM.pdf]

Supplementary Table 1: Quality control and Quality assurance SNP filtering for the two Saudi MI studies, CardioGRAMplusC4D and the UK BioBank GWAS for 60,228 MI cases and 580,040 non-MI controls.

	Variants	Variants $p < 5 \times 10^{-8}$	Variants $P(R) < 5 \times 10^{-8}$
CARDIoGRAMplusC4D+UKBB	8,126,033	4,419	
Saudi MI Study 1& 2 Meta-Analysis	11,819,213	31	19
Saudi MI Study 1& 2 Meta-Analysis (I2 < 50)	10,128,822	28	19
CARDIoGRAMplusC4D/UKBB + Saudi MI Study 1& 2 Meta-Analysis	7,036,716	3,701	902
CARDIoGRAMplusC4D/UKBB + Saudi MI Study 1& 2 Meta-Analysis (I2 <50)	5,889,786	1,636	902
*Number of SNPs for each dataset where comparison associations were found between all input data. Saudi MI Study 1& 2 Meta-Analysis = 3,950 cases and 2,324 controls CARDIoGRAMplusC4D/UKBB + Saudi MI Study 1& 2 = 60,228 MI cases and 580,040 controls.			
